# Nutrient supplementation among pregnant women in China: an observational study

**DOI:** 10.1017/S1368980021001269

**Published:** 2022-06

**Authors:** Tao Han, Jingwen Dong, Jiangtao Zhang, Chenxiao Zhang, Yuxuan Wang, Zhiruo Zhang, Mi Xiang

**Affiliations:** School of Public Health, Shanghai Jiao Tong University, Shanghai 200025, People’s Republic of China

**Keywords:** Nutrient supplementation, Pregnant women, Primary source of information, China

## Abstract

**Objective:**

To clarify nutrient supplementation usage and primary source of information among pregnant women in China.

**Design:**

This cross-sectional study used information on nutrient supplementation and primary source of information collected via face-to-face interviews. Data on the usage of folic acid, Ca/vitamin D, Fe, vitamins, DHA and other dietary supplements were collected. Primary source of information was categorised as family/relatives, friends/co-workers, the Internet, books/magazines, television/radio, doctors, other people and oneself.

**Setting:**

Maternal and Child Health Hospital in Chengdu, China.

**Participants:**

One thousand eighty-one Chinese pregnant women aged ≥20 years with singleton pregnancies.

**Results:**

In all three trimesters of pregnancy, usage was highest and most stable for folic acid (81·7 %), followed by vitamins (vitamin A, B-group vitamins, vitamin C and multivitamins; 75·0 %), whereas Ca/vitamin D (51·4 %) and Fe (18·1 %) usage was low, potentially indicating a deficiency risk. All supplementation usage percentages increased with pregnancy duration (*P* < 0·05). Notably, approximately 10 % of the pregnant women in our study did not use any nutrient supplementation, and this was especially common in early pregnancy. More than 50 % of the women reported getting information on nutrient supplementation from family members, and about 30 % reported getting this information from doctors.

**Conclusions:**

Among pregnant women in China, awareness about nutrient supplementation increases as the pregnancy progresses, but some types of nutrient supplementation (such as Ca/vitamin D and Fe) remain at low levels. It is necessary to pay more attention to the health education of pregnant women in China, and the influence of family members should be emphasised.

Micronutrient intake is essential for fetal development and for the health of pregnant women^(1)^. Examples of effective single micronutrient interventions include folic acid to prevent neural tube defects, Fe to prevent anaemia, and Ca and vitamin D to reduce the risk of low birth weight^(2,3)^. However, micronutrient deficiencies during pregnancy remain widespread globally^(4)^. In Italy, both pregnant and lactating women were found to have inadequate intake of Fe, Ca, folic acid and vitamin D^(5)^ and Indonesian pregnant women’s intake of Fe, Ca and vitamins was found to be below locally recommended levels^(6)^. A systematic review of data from Ethiopia, Kenya, Nigeria and South Africa revealed that pregnant women in Africa had vitamin A, iodine, Zn and folic acid deficiencies^(7)^. One potential reason for these deficiencies is that pregnant women may have difficulty meeting all their nutritional needs through diet alone. During pregnancy, nutrient requirements increase to adapt to fetal needs. Indeed, most women in Canada have been found to have dietary intakes below the estimated average requirements for micronutrients such as Fe, vitamin D and folic acid^(8)^. A study collecting 24-h dietary recall data in eight cities in China (2011–2012) reported that the mean intakes of folic acid, Ca, Fe and vitamins were below the recommended nutrient intake and estimated average requirements^(9)^.

Using nutrient supplementation during pregnancy can alleviate this harmful situation and has been demonstrated to be an important method of reducing the risk of inadequate nutrient intake. The supplementation of nutrients such as Fe and folic acid has been proven to reduce the risk of puerperal sepsis, maternal anaemia and preterm birth^(2)^. Inadequate Ca intake can cause preeclampsia in pregnant women^(3)^. Vitamins and other supplements, such as iodine and Zn, have also been shown to potentially have positive effects, although this remains unclear^(10)^. The WHO has published several recommendations regarding the use of vitamins and supplements^(2,2,3,11)^.

Understanding of the situation regarding nutrient supplementation can help governments and hospitals to counteract nutritional deficiencies and improve pregnancy outcomes through implementing relevant interventions and efficient policies^(4)^. However, the use of nutrient supplementation among pregnant women in China has not yet been clarified; this study is the first to provide a clear and comprehensive survey of the use of various nutrient supplementations and the sources of information about nutrient supplementation among pregnant women in China. By doing this, we hope to lay a foundation for future nutrition education and interventions.

## Methods

### Study design and sample

A cross-sectional survey was conducted through face-to-face interviews conducted from June 2014 to January 2016. A total of 1081 participants were recruited from the Maternal and Child Health Hospital in Chengdu Wuhou in the Sichuan province of western China. The inclusion criteria were being over the age 20 years and having a singleton pregnancy. Women with any major chronic disease (diabetes mellitus, hypertension, heart disease, chronic renal disease or other diseases) were excluded. A total of 178 respondents were excluded from the analysis because of missing data; the remaining 903 respondents were categorised by trimester (first trimester: 1–12 weeks, second trimester: 13–27 weeks, third trimester: 28–40 weeks). In terms of primary source of information about nutrient supplementation, additional 163 participants were excluded because of missing data of information about nutrient supplementation and demographic variables, but there was no statistical difference between the excluded samples and the remaining samples. All eligible participants who consented to participate were informed about the study’s purpose and procedures and were asked to complete the survey. If the participants had questions during the survey, they were able to ask the investigators. This study was approved by the Committee on Research Involving Human Subjects of Waseda University (approval number: 2014-037), and all participants provided written informed consent.

### Sociodemographic variables

The collected sociodemographic information included age, pregnancy trimester, marital status, education level, employment status and household income. Age was categorised into three groups: <25 years, 25–29 years and ≥30 years. Pregnancy trimester was categorised as the first, second or third trimester. Marital status was categorised as unmarried or married. Education level was categorised as <high school degree, high school degree or >high school degree. Employment status was categorised as unemployed or employed, and monthly household income was categorised as <4000, 4000–8000 or >8000 Chinese yuan.

### Data on nutrient supplementation and primary source of information

Data on nutrient supplementation during pregnancy were collected during the survey. Participants were asked about their usage of folic acid, Ca/vitamin D, Fe, vitamin A, B-group vitamins, vitamin C, multivitamins, DHA and other dietary supplements. Participants were then asked their primary source of information on nutrient supplementation.

Following the WHO guidelines^(2,11)^, vitamin A, B-group vitamins and vitamin C were classified as ‘vitamins’ and DHA and other dietary supplements were classified as ‘other supplements’ in the descriptive analysis. The number of types of nutrient supplementation was derived by summing the originally reported specific nutrient supplements. Each woman’s primary source of nutrient supplementation information was categorised as family/relatives, friends/co-workers, the Internet, books/magazines, television/radio, doctors, other people or oneself.

### Statistical analyses

All statistical analyses were performed using SPSS Statistics, version 22.0. Frequencies and percentages were calculated for categorical data. To examine differences across the three trimesters, we divided the data by trimester, creating three data sets, and descriptive statistics were calculated separately for each data set. Additionally, *χ* ^2^ tests were used to examine differences in nutrient supplementation over the three trimesters. The sample size varied for some analyses because of missing data on covariates, and this is indicated in table footnotes. Statistical significance was set at *P* < 0·05.

## Results

A total of 903 pregnant women were enrolled in this study. Table 1 presents the sociodemographic characteristics of all the participants. Their mean age was 27 ± 4 years. In terms of trimester, 287 women (31·8 %) were in the first trimester (mean: 9·2 ± 3·5 weeks), 307 (34·0 %) were in the second trimester (mean: 19·9 ± 5·9 weeks) and 309 (34·2 %) were in the third trimester (mean: 19·4 ± 8·6 weeks). There were no differences in sociodemographic characteristics by trimester. Among all participants, 93·5 % were married and 47·3 % had a high school education or less. Further, 56·7 % of the participants were unemployed, and only 25·1 % had household incomes >8000 Chinese yuan.

**Table 1:** Sociodemographic characteristics of the survey participants (*n* 903 pregnant women)

	*n*	%
Age (years)		
<25	255	28·8
25–29	448	50·7
≥30	181	20·5
Trimester		
First Trimester	287	31·8
Second Trimester	307	34·0
Third Trimester	309	34·2
Marital status		
Unmarried	57	6·5
Married	825	93·5
Education level		
<High school degree	176	19·7
High school degree	247	27·6
>High school degree	472	52·7
Employment status		
No work	512	56·7
Working	391	43·3
Household income		
<¥ 4000	245	28·1
¥ 4000–8000	407	46·7
>¥ 8000	219	25·1

**Fig. 1:**
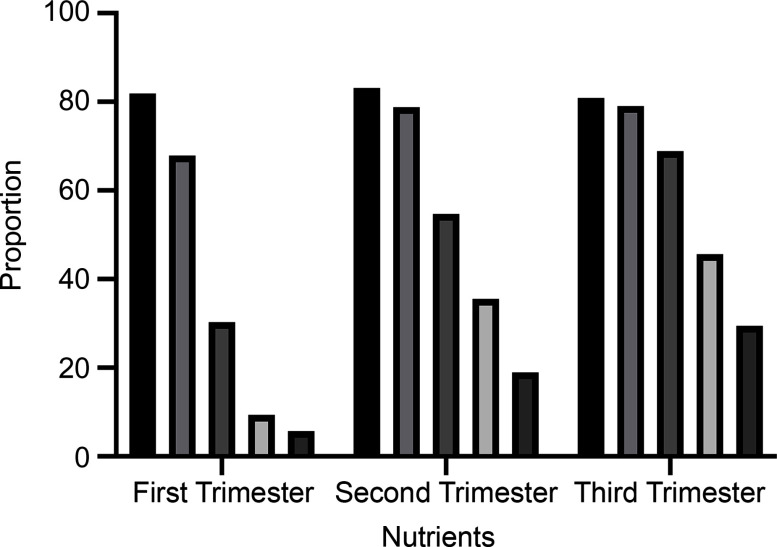
Nutritional status of pregnant women by trimester. 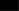, folic acid; 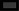, calcium/Vitamin D; 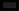, iron; 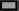, vitamins; 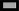, other supplements

Nutrient supplementation during pregnancy varied by trimester, as is shown in detail in Table 2. In each trimester, more than four-fifths (81·9 % in the first trimester, 83·1 % in the second trimester and 80·9 % in the third trimester) of the pregnant women took folic acid, with an overall average of 81·7 %. The percentage of women taking Ca/vitamin D was 30·3 % in the first trimester, 54·7 % in the second trimester and 68·9 % in third trimester. An overall average of 18·1 % of the pregnant women reported taking Fe (5·6 % in the first trimester, 18·9 % in the second trimester and 29·4 % in the third trimester). As shown in Fig. 1, the use of Ca/vitamin D, Fe, vitamins and other supplements significantly increased with pregnancy trimester.

**Table 2:** Use of nutrient supplementation among pregnant women by trimester (*n* 903)

	Overall		First trimester		Second trimester		Third trimester		*χ* ^2^ test*
	(*n* 903)		(*n* 287)		(*n* 307)		(*n* 309)		
	*n*	%	*n*	%	*n*	%	*n*	%	
Folic acid	740	81·7	235	81·9	255	83·1	250	80·9	0·785
Ca/Vitamin D	468	51·4	87	30·3	168	54·7	213	68·9	**< 0·001**
Fe	165	18·1	16	5·6	58	18·9	91	29·4	**< 0·001**
Vitamins†	681	75·0	195	67·9	242	78·8	244	79·0	**0·002**
Other supplements†	277	30·4	27	9·4	109	35·5	141	45·6	**< 0·001**

* *P*-values are from the *χ* ^2^ test. *P*-value < 0·05 (bold) indicates a statistically significant difference.

† Vitamin A, B-group vitamins, vitamin C and multivitamins are combined into the ‘vitamins’ category. DHA and other dietary supplements are combined into ‘other supplements’.

Table 3 presents the number of types of nutrient supplementation used over the three trimesters of pregnancy. The percentage of all participants who used two or more types of nutrient supplementation was 79·73 %, and 10·74 % used no nutrient supplementation. The percentage of participants who used two or more kinds of nutrient supplementation increased significantly as the pregnancy progressed (69·34 % in the first trimester, 83·06 % in the second trimester and 86·08 % in the third trimester; *P* < 0·001). Additionally, the percentage of participants using no supplementation declined as the pregnancy progressed (15·33 % in the first trimester, 10·10 % in the second trimester and 7·12 % in the third trimester; *P* = 0·005).

**Table 3:** Number of types of nutrient supplementation used among pregnant women by trimester (*n* 903)*

	Overall		First Trimester		Second Trimester		Third Trimester		*χ* ^2^ test
	(*n* 903)		(*n* 287)		(*n* 307)		(*n* 309)		
	*n*	%	*n*	%	*n*	%	*n*	%	
0 kind	97	10·74	44	15·33	31	10·10	22	7·12	0·005
1 kind	86	9·52	44	15·33	21	6·84	21	6·80	0·000
≥2 kinds	720	79·73	199	69·34	255	83·06	266	86·08	0·000

* The nutrient supplementation categories are folic acid, Ca/vitamin D, Fe, vitamin A, B-group vitamins, vitamin C, multivitamins, DHA, and other dietary supplements.

**Fig. 2:**
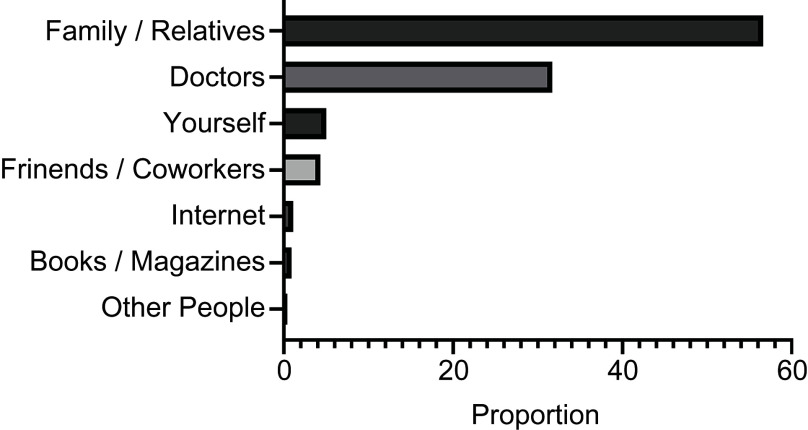
Sources of information about nutrient supplementation for pregnant women

Table 4 shows information on the women’s primary source of information about nutrient supplementation during pregnancy. Over half of the pregnant women in our sample received information about nutrient supplementation from family members and relatives, and approximately 30 % of the women received this type of information from doctors. These results were consistent across all three trimesters. Information sources other than family members/relatives and doctors were used by only about 15 % of the survey respondents. Figure 2 shows that the most frequently used source of information about nutrient supplementation for pregnant women was family/relatives, followed by doctors, themselves, colleagues/friends, the Internet, books/magazines and other people.

**Table 4:** Primary source of information about nutrient supplementation during pregnancy by trimester (*n* 740)*

	First trimester		Second trimester		Third trimester	
	(*n* 235)		(*n* 255)		(*n* 250)	
	*n*	%	*n*	%	*n*	%
Sources						
Family/Relatives	141	60·0	137	53·7	144	57·6
Doctors	68	28·9	80	31·4	85	34·0
Yourself	9	3·8	14	5·5	13	5·2
Friends/Co-workers	10	4·3	17	6·7	5	2·0
Internet	5	2·1	3	1·2	0	0·0
Books/Magazines	2	0·9	4	1·6	1	0·4
Other people	0	0·0	0	0·0	2	0·8

* A total of 163 participants were excluded because of the missing data on primary source of information about nutrient supplementation.

## Discussion

This study was the first to provide a comprehensive survey of the current use of nutrient supplementation and the sources of information about nutrient supplementation among pregnant women in China. The study revealed that, although most pregnant women in China reported taking folic acid and vitamins during pregnancy, which was a positive finding, the use of Ca/vitamin D and Fe supplementation was low, potentially indicating a risk of deficiency. The trimester of pregnancy was positively associated with all types of nutrient supplementation. Women in early pregnancy require special attention because of their potentially insufficient use of nutrient supplementation. Family members and relatives are an important source of information, and health education should be strengthened.

We found that the utilisation rate of folic acid was the highest among various types of nutrient supplementation, and this was stable across all pregnancy trimesters. This finding was similar to the survey results reported by Qin showing that 86·1 % of pregnant women in China used folic acid^(12)^. Folic acid, which has been widely studied, can prevent neural tube defects, and a folic acid deficiency can increase the risk of convulsions and fetal malformations^(10,13,14)^. Adherence to folic acid supplementation has also been found to be high among pregnant women in India, where the most common cause of noncompliance was forgetfulness^(15)^. Vitamins have also attracted a great deal of attention in recent years. Notably, however, the use of Ca/vitamin D and Fe supplementation was low in our research, which may lead to insufficiencies in Ca/vitamin D and Fe and, ultimately, contribute to negative outcomes among pregnant women in China. Previous studies of Chinese pregnant women reported that the mean intakes of Ca/vitamin D and Fe were below the recommended nutrient intake and estimated average requirement^(9)^ and that the prevalence of Fe deficiency was as high as 48·16 %, with 13·87 % of Chinese pregnant women suffering from Fe-deficient anaemia^(16)^. Vitamin D deficiency is more pronounced in some other countries. One study found that the Vitamin D deficiency rate of pregnant women in Saudi Arabia was 94·2 %^(17)^. Vitamin D deficiency may be related to obesity and insufficient exposure to sunlight^(18)^. Other studies have shown an increase in the prevalence of hypertensive disorders of pregnancy, which is among the top three leading causes of death among pregnant women and is closely related to Ca deficiency^(19)^.

Ca/vitamin D and Fe are important during pregnancy. Previous studies have shown that newborns whose mothers were deficient in Ca and vitamin D had significantly lower birth weight, body length and head circumference^(20)^ and even lower neurodevelopmental levels^(21)^ than did newborns born to mothers with normal levels of Ca and vitamin D. Furthermore, the WHO has reported that Fe deficiency can affect fetal growth and increase the risk of premature birth, low birth weight and postpartum bleeding^(2)^. Although China’s *National Standard for Food Safety* lists Fe and vitamin D as essential ingredients to include in dietary supplements for pregnant women, awareness regarding Ca/vitamin D and Fe supplementation remains relatively low and has not increased for many years^(22)^. Thus, appropriate educational or other interventions to increase Ca/vitamin D and Fe intake among pregnant women are important and must be emphasised in the future. Additionally, although most of the pregnant women in our study used two or more kinds of nutrient supplementation throughout their pregnancy, approximately 10 % of these women did not use any kind of nutrient supplementation. The lack of nutrient supplementation was highest among women in the first trimester of pregnancy, 15 % of whom reported no nutrient supplement use. Thus, women in early pregnancy should also receive special attention.

Our results revealed that nutrient supplementation awareness among pregnant women differed significantly by trimester. The use of Ca/vitamin D, Fe, vitamins and other supplements increased as the pregnancy progressed. As was also found in a previous study in Canada, nutrient supplementation usage was similar across the three trimesters of pregnancy, except for vitamin D, which was observed to be deficient in early pregnancy^(23)^. Thus, nutrient supplementation awareness was lowest in early pregnancy for the Chinese women in the present study, which may mean that extra attention should be directed towards women in early pregnancy to prevent nutrient deficiency. Increasing evidence suggests that micronutrient supplementation is important in early pregnancy because nutritional deficiencies during this period can adversely affect the placental structure and, indirectly, birth weight^(24)^. The effects of fetal nutrition may persist well into adulthood, with possible intergenerational effects^(25)^. For example, folic acid is integral in the synthesis of DNA and neurotransmitters, and Ca is an essential nutrient for bone mineralisation and cell membrane maintenance^(13,26)^. To better carry out health education regarding meeting the demand for micronutrients during pregnancy, more interventions should be implemented to reduce the proportion of women at high risk of nutrient deficiency, particularly in the first trimester of pregnancy.

This study was the first to investigate primary source of information about nutrient supplementation among pregnant women in China, and some of the findings are particularly notable. More than 85 % of the surveyed pregnant women reported receiving information about nutrient supplementation from family members/relatives or doctors, with more than half of the women reporting family members and relatives as a source of information. This large percentage indicates that family members play an important role in motivating pregnant women to take the necessary nutrient supplementation. Clearly, family-centred health education should be used with the family members of pregnant women. Our findings also revealed that pregnant women appear to receive relatively little nutrition information from the Internet, books, magazines or other types of people, which may stem from a distrust of information from these sources. In contrast, a systematic review of thirty-one studies from fourteen countries showed that the most frequent information source used by women during pregnancy was health professionals, followed by informal sources (family and friends) and the Internet. This difference may be attributed to the fact that pregnant women in China tend to feel ashamed or embarrassed about talking about pregnancy-related issues with strangers^(27)^. Thus, increased trust in the doctor–patient relationship and improved accuracy of information are needed to provide proper prenatal care and nutritional guidance for women during pregnancy.

Our study has several limitations. First, although we chose a large region with rural and urban areas and a population of approximately 16 million, the participants were recruited from only one city, which may make our findings less generalisable. Second, because this was a cross-sectional study, causality could not be established. Despite these limitations, our study also has strengths in several aspects. First, although the use of nutrient supplementation and sources of information about nutrient supplementation were self-reported by the pregnant women, we collected the data through face-to-face interviews, which are characterised by high credibility and validity. In terms of the research design, our study had a large sample of pregnant women across all three trimesters. These strengths allowed the study to provide a comprehensive understanding of pregnant women’s nutrient supplementation, whereas previous studies in China have focused only on nutrient intake through food, ignoring nutrient supplementation. Our study also covered a rich variety of nutrient supplementation, and we additionally evaluated pregnant women’s sources of information about nutrient supplementation, which has not been examined in any previous studies. This distinctive quality made it possible for us to provide a theoretical basis for future interventions related to pregnant women’s nutrient supplementation.

## Conclusions

In China, pregnant women’s awareness of the importance of nutrient supplementation increases as the pregnancy progresses. Because important types of micronutrient supplementation (Ca/vitamin D and Fe supplementation) remain at low levels, especially in early pregnancy, it is necessary to pay more attention to health education and other interventions to increase nutrient supplementation among pregnant women in China. Additionally, the influence of pregnant women’s family members on their nutrient supplementation should be emphasised in health education and other initiatives.

## References

[1] Groth SW , Stewart PA , Ossip DJ et al. (2017) Micronutrient intake is inadequate for a sample of pregnant African-American women. J Acad Nutr Diet 117, 589–598. 28065633 10.1016/j.jand.2016.11.011 PMC5367978

[2] World Health Organization (2012) Daily Iron and Folic Acid Supplementation in Pregnant Women. Geneva: WHO. 23586119

[3] World Health Organization (2013) Calcium Supplementation in Pregnant Women. Geneva: WHO. 24006556

[4] Gernand AD , Schulze KJ , Stewart CP et al. (2016) Micronutrient deficiencies in pregnancy worldwide: health effects and prevention. Nat Rev Endocrinol 12, 274–289. 27032981 10.1038/nrendo.2016.37 PMC4927329

[5] Marangoni F , Cetin I , Verduci E et al. (2016) Maternal diet and nutrient requirements in pregnancy and breastfeeding. An Italian consensus document. Nutrients 8, 629. 27754423 10.3390/nu8100629 PMC5084016

[6] Madanijah S , Briawan D , Rimbawan R et al. (2016) Nutritional status of pre-pregnant, pregnant women residing in Bogor district, Indonesia: a cross-sectional dietary, nutrient intake study. Br J Nutr 116, S57–S66. 27079653 10.1017/S000711451600057X

[7] Harika R , Faber M , Samuel F et al. (2017) Micronutrient status and dietary intake of iron, vitamin A, iodine, folate and zinc in women of reproductive age and pregnant women in Ethiopia, Kenya, Nigeria and South Africa: a systematic review of data from 2005 to 2015. Nutrients 9, 1096. 28981457 10.3390/nu9101096 PMC5691713

[8] Dubois L , Diasparra M , Bédard B et al. (2017) Adequacy of nutritional intake from food and supplements in a cohort of pregnant women in Québec, Canada: the 3D Cohort Study (Design, Develop, Discover). Am J Clin Nutr 106, 541–548. 28615265 10.3945/ajcn.117.155499

[9] Liu FL , Zhang YM , Pares GV et al. (2015) Nutrient intakes of pregnant women and their associated factors in eight cities of China: a cross-sectional study. Chin Med J 128, 1778–1786. 26112720 10.4103/0366-6999.159354 PMC4733713

[10] Mousa A , Naqash A & Lim S (2019) Macronutrient and micronutrient intake during pregnancy: an overview of recent evidence. Nutrients 11, 443. 30791647 10.3390/nu11020443 PMC6413112

[11] World Health Organization (2011) Vitamin A Supplementation in Pregnant Women. Geneva: WHO.

[12] Qin YW , Fan XX , Lu YP et al. (2014) Analysis on folic acid usage status of pregnancy women in Sichuan province, rural – urban differences. Chin J Family Planning, Gynecotokol 6, 69–71.

[13] De-Regil LM , Pena-Rosas JP , Fernandez-Gaxiola AC et al. (2015) Effects, safety of periconceptional oral folate supplementation for preventing birth defects. Cochrane Database Syst Rev 12, CD007950. 10.1002/14651858.CD007950.pub3 PMC8783750 26662928

[14] Berry RJ , Li Z , Erickson JD et al. (1999) Prevention of neural-tube defects with folic acid in China. China-U.S. collaborative project for neural tube defect prevention. N Engl J Med 341, 1485–1490. 10559448 10.1056/NEJM199911113412001

[15] Debi S , Basu G , Mondal R et al. (2020) Compliance to iron-folic-acid supplementation and associated factors among pregnant women: a cross-sectional survey in a district of West Bengal, India. J Fam Med Prim Care 9, 3613–3618. 10.4103/jfmpc.jfmpc_392_20 PMC7567271 33102338

[16] He GL , Sun X , Tan J et al. (2018) Survey of prevalence of iron deficiency and iron deficiency anemia in pregnant women in urban areas of China. Zhonghua Fu Chan Ke Za Zhi 53, 761–767. 30453423 10.3760/cma.j.issn.0529-567x.2018.11.006

[17] Al-Ajlan A , Krishnaswamy S , Alokail MS et al. (2015) Vitamin D deficiency and dyslipidemia in early pregnancy. BMC Pregnancy Childbirth 15, 314. 26610599 10.1186/s12884-015-0751-5 PMC4662014

[18] Ardawi MS , Qari MH , Rouzi AA et al. (2011) Vitamin D status in relation to obesity, bone mineral density, bone turnover markers and vitamin D receptor genotypes in healthy Saudi pre- and postmenopausal women. Osteoporosis Int 22, 463–475. 10.1007/s00198-010-1249-7 20431993

[19] Dong CX & Yin SA (2018) The ten-year retrospect of nutrition and health status of pregnant women in China. Zhonghua Yu Fang Yi Xue Za Zhi 52, 94–100. 29334717 10.3760/cma.j.issn.0253-9624.2018.01.019

[20] Song SJ , Si S , Liu J et al. (2013) Vitamin D status in Chinese pregnant women and their newborns in Beijing and their relationships to birth size. Public Health Nutr 16, 687–692. 23174124 10.1017/S1368980012003084 PMC10271381

[21] Chi MZ , Zhu L , Jin FF et al. (2017) Brief research of vitamin D level in pregnant women and its influence on infantile neurodevelopment. China Pract Med 12, 25–27.

[22] Wang J , Zhao LY , Piao JH et al. (2011) Nutrition and health status of pregnant women in 8 provinces in China (in Chinese with English Abstract). J Hyg Res 40, 201–203. 21560309

[23] Savard C , Lemieux S , Weisnagel SJ et al. (2018) Trimester-specific dietary intakes in a sample of French-canadian pregnant women in comparison with National Nutritional Guidelines. Nutrients 10, 768. 29899222 10.3390/nu10060768 PMC6024697

[24] Luke B (1994) Nutritional influences on fetal growth. Clin Obstet Gynecol 37, 538–549. 7955642 10.1097/00003081-199409000-00007

[25] Anderson AS (2001) Symposium on ‘nutritional adaptation to pregnancy and lactation’. Pregnancy as a time for dietary change? Proc Nutr Soc 60, 497–504. 12069403 10.1079/pns2001113

[26] Buppasiri P , Lumbiganon P , Thinkhamrop J et al. (2015) Calcium supplementation (other than for preventing or treating hypertension) for improving pregnancy and infant outcomes. Cochrane Database Syst Rev 2, CD007079. 10.1002/14651858.CD007079.pub2 21975761

[27] Ghiasi A (2019) Health information needs, sources of information, and barriers to accessing health information among pregnant women: a systematic review of research. J Matern Fetal Neonatal Med 34, 1320–1330. doi:10.1080/14767058.2019.1634685 31216921

