# Nutrient supplementation among pregnant women in China: an observational study – ERRATUM

**DOI:** 10.1017/S1368980023000290

**Published:** 2023-05

**Authors:** Tao Han, Jingwen Dong, Jiangtao Zhang, Chenxiao Zhang, Yuxuan Wang, Zhiruo Zhang, Mi Xiang

Cambridge University Press & Assessment apologise for an error in the above article. Mi Xiang was credited as the sole corresponding author.

Zhiruo Zhang is the co-corresponding author. This has been updated.

## References

[ref1] Han, T. , Dong, J. , Zhang, J. , Zhang, C. , Wang, Y. , Zhang, Z. , & Xiang, M. (2022). Nutrient supplementation among pregnant women in China: An observational study. Public Health Nutrition, 25(6), 1537–1542. doi: 10.1017/S1368980021001269 33749574 PMC9991596

